# Choriocapillaris flow deficit is associated with disease duration in type 2 diabetic patients without retinopathy: a cross-sectional study

**DOI:** 10.1186/s40942-024-00611-y

**Published:** 2024-11-26

**Authors:** Lourdes Vidal-Oliver, Elisa Herzig-de Almeida, Sabrina Spissinger, Robert P Finger

**Affiliations:** Department of Ophthalmology, University Hospital Mannheim & Medical Faculty Mannheim, University of Heidelberg, Theodor-Kutzer-Ufer 1-3, Mannheim, 68167 Germany

**Keywords:** Choriocapillaris flow deficit, Diabetes mellitus, Duration of the disease, Optical coherence tomography angiography

## Abstract

**Background:**

Diabetes mellitus (DM) causes microvascular damage due to long-term hyperglycemia, even before the onset of retinal changes. We aimed to investigate the association between optical coherence tomography angiography (OCTA) metrics and disease duration in type 2 diabetic patients without retinopathy.

**Methods:**

Eighty-two eyes of 82 type 2 diabetic patients without diabetic retinopathy (DR) were included. Choriocapillaris flow deficit (CC FD%), vessel density (VD), vessel length density (VLD) in the superficial (SVP) and deep vascular plexus (DVP) were calculated and compared between different sectors of the macula. Foveal avascular zone circularity (FAZc) was also calculated. Linear regression was used to study the association of each vascular parameter with disease duration both in a univariate and multivariate design adjusting for age, sex, Hb1Ac and arterial hypertension.

**Results:**

CC FD% increased by 3.7, 2.3, 3.8 and 4.6% in the nasal, superior, temporal and inferior sectors per decade of disease duration, after adjusting for confounders. Mean values of VD and VLD in the SVP and DVP, as well as FAZc decreased with increased duration of DM, but the association was weaker. Only the VD in the superior and temporal sectors of the SVP were significant in the multivariate analysis (ß=-0.12 (95% CI -0.24 to -0.01) and − 0.13 (95%CI -0.25 to -0.002), respectively).

**Conclusion:**

CC FD% is independently associated with disease duration in type 2 diabetes independent of the presence of clinical retinopathy. Further longitudinal studies are needed to investigate the role of choroidal changes in predicting DR onset in order to individualize screening protocols.

## Background

Diabetes mellitus type 2 (T2DM) is one of the most prevalent chronic diseases in the general population with a potential risk for sight-threatening complications. A recent meta-analysis found that diabetic eye disease occur in approximately 25% of cases, with an estimated 8.6 million individuals in Europe potentially affected by 2050 [1]. Diabetic retinopathy (DR) is characterized by microvascular changes caused by persistent hyperglycemia, but macroscopically inapparent tissue damage precedes these disease-defining changes. Monitoring these microscopic changes holds opportunity for DR screening and secondary prevention approaches which are increasingly needed in light of the ever growing screening burden in T2DM.

Modern diagnostics assessing retinal function or microanatomy already showed alterations in diabetic patients without or before onset of clinically visible retinopathy [2, 3]. Optical coherence tomography angiography is of particular interest for monitoring retinal microanatomy because it allows rapid and non-invasive three-dimensional imaging of the retinal vasculature – the site of pathophysiological changes. En face OCTA projections permit quantifying the different retinal plexus using various metrics, down to the choriocapillaris in high quality images [4]. 

To date, it is unclear which OCTA parameters should be considered as outcome parameters. To identify relevant OCTA metrics we explored the association of different quantifiable OCTA parameters with disease duration, the main risk factor for DR onset [5]. 

## Methods

In this single-center, cross-sectional study, we recruited adult patients diagnosed with T2DM without diabetic retinopathy. This study was conducted in accordance with the Declaration of Helsinki and was approved by the Institutional Review Board of the Medical Faculty of Mannheim (ID number: 2023 − 666). Written informed consent was obtained before participation.

We screened 123 type 2 diabetic patients who presented to the ophthalmology or endocrinology clinic of a tertiary referral center. The study included patients with T2DM and without diabetic retinopathy in any eye, after review of 5-field color fundus photography. Optical coherence tomography (OCT), and OCT angiography (OCTA) were also performed. Exclusion criteria were: low-quality OCT and OCTA images (defined as a signal-to-noise ratio (Q-value) < 30dB or with significant image artifacts: attenuation, striping, defocus or projection), presence of any retinopathy, glaucoma or significant media opacities. When both eyes of the same patient met inclusion criteria, the eye with the best image quality defined by a retina specialist was selected.

We excluded 31 patients due to presence of clinically visible diabetic retinopathy seen on fundus examination, 5 patients for having concomitant age-related macular degeneration and 5 additional patients due to poor image quality in both eyes, leaving 82 patients for further analysis.

Primary outcome measures were duration of the diabetes (in years) and its association with vascular OCTA parameters, including vessel density (VD), vessel length density (VLD), choriocapillaris flow deficit area (CC FD%) and foveal avascular zone (FAZ) circularity. Exploratory outcomes included associations with arterial hypertension, age and sex. Patients taking antihypertensive medication were defined as having arterial hypertension.

### Image acquisition

We acquired macular OCT images with a Spectral-Domain OCT Spectralis HRA-OCT3 (Heidelberg Engineering, Heidelberg, Germany), with 18 averaged images by automatic real time function, a 122 spacing between B-scans and at 85 kHz scan speed.

OCTA imaging protocol included a 20 × 20º macular scan at 85 kHz A-scan rate and a spacing of 11 microns between B-scans.

### Image processing and analysis

En face projections of OCTA images at the superficial (SVP), intermediate (ICP) and deep (DVP) vascular plexus and the choriocapillaris (CC) were exported in a 1:1 pixel scale and imported into ImageJ^®^ (version 2.14.0/1.54f, National Institutes of Health, Bethesda, MD, USA). The segmentation boundaries used to define the different retinal plexuses were those preset by the HEYEX software (Heidelberg Engineering, Heidelberg, Germany). The SVP contained the ganglion cell layer and the inner plexiform layer; the ICP was situated between inner plexiform layer and inner nuclear layer, the DVP between inner and outer plexiform layer and the CC 10 μm below the Bruch’s membrane.

**Fig. 1:**
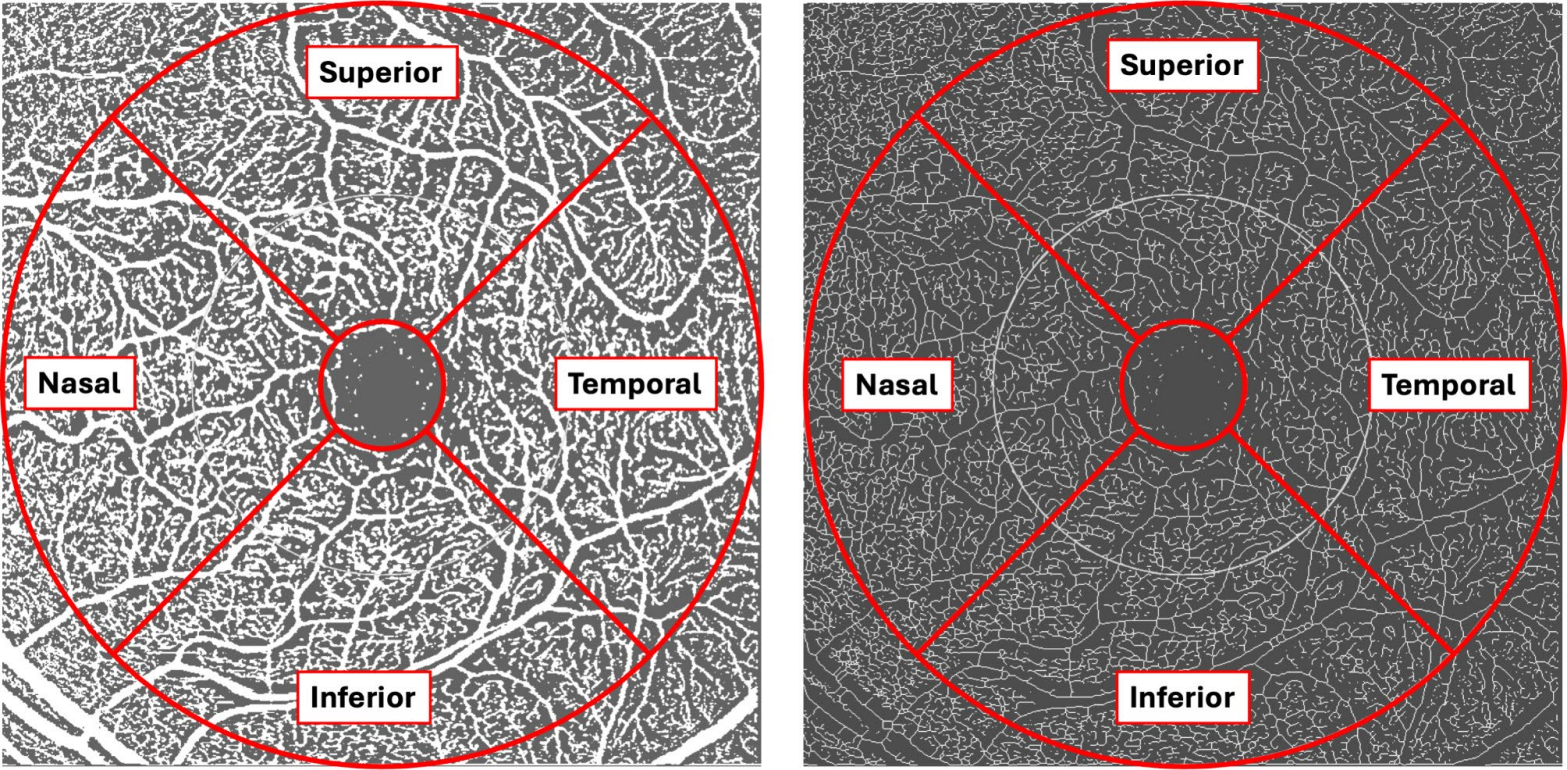
En face OCTA scan of the superficial vascular plexus after binarization and skeletonization. The nasal, superior, temporal and inferior sectors of the ETDRS grid centered on the foveal avascular zone we included in the analysis are depicted

Using imageJ^®^, images were first binarized with the Phansalkar Auto Local Threshold method (radius = 15) and an ETDRS grid was added centered on fovea [6, 7]. Vessel density (VD, defined as white pixels/image area, %) was calculated in the binarized images in both the SVP and DVP in the nasal, superior, temporal and inferior sectors of the ETDRS grid (Fig. 1). Subsequently, images were skeletonized to calculate vessel length density in the same sectors (VLD, defined as vessel length per unit area, mm^− 1^).

CC flow deficit percentage (CC FD, %) was automatically calculated from normalized and then binarized images of the choriocapillaris slab, using the Phansalkar’s local thresholding method, as previously described [8]. CC FD% was then calculated as area without flow signal/total area, using the Analyze Particles tool. We excluded those areas < 24microns as this is equivalent to the normal intercapillary distance [9]. CC FD% was calculated in the same 4 sectors of the ETDRS grid (nasal, superior, temporal and inferior), centered on the same coordinates as the corresponding SVP and DVP images.

The area (mm^2^) and perimeter (mm) of the FAZ were measured manually using the freehand selection tool in en face images from the ICP, as previous authors have reported a slightly better repeatability of FAZ measurements on this plexus [10]. The circularity of the FAZ was then calculated using the following formula: 4π*area/perimeter^2^ (unitless), as previously described. FAZ circularity values range from 0 to 1, with a value of 1 representing a perfect circle. We analyzed only FAZ circularity because it showed less interindividual variability than FAZ area and perimeter [11, 12]. 

Peripheral areas were not included in the analysis to avoid bias due to image attenuation.

### Statistical analysis

Reported descriptive statistics include mean values and standard deviation. The ANOVA test was used to compare OCTA metrics between the different areas of the retina studied after assessing the normality of the distribution using the Shapiro-Wilk test.

Linear regression was used to study association between duration of the disease (dependent variable) and OCTA vascular metrics: for each of the retinal sectors (nasal, superior, temporal and inferior) including either VD, VLD, FAZc and CC FD%.

For each variable showing significant association with disease duration in the univariate analysis, a multivariate analysis was performed to adjust the model for the following confounders: age, sex, Hb1Ac and the presence of arterial hypertension.

All analysis were performed using GraphPad Prism^®^ (version 10.2.1).

## Results

We included 82 patients, of whom one eye each was included in further analyses. Patients were on average 59.5 years (range 24–86), with a mean T2DM duration of 9.5 years (range 0.2–40) and glycemic control of HbA1c 7.01 ± 1.76%. Clinical and demographic characteristics of the patients included are shown in Table 1.

**Table 1:** Demographic and clinical characteristics of the cohort

Patient characteristics *N* = 82	
Age (years)	59.5 (range 24–86)
Sex (M/F; N, %)	45/37 ( 54.9/45.1%)
Disease duration - years (SD)	9.48 (SD 8.71)
Arterial Hypertension (N,%)	65 (79.3%)
Best-corrected Visual Acuity (LogMAR)	-0.02 (range − 0.1–0.2)
Spherical error (diopters)	-0.26 (range − 5.75 to + 4)
HbA1c (%)	7.01 (SD 1.76)

**Fig. 2:**
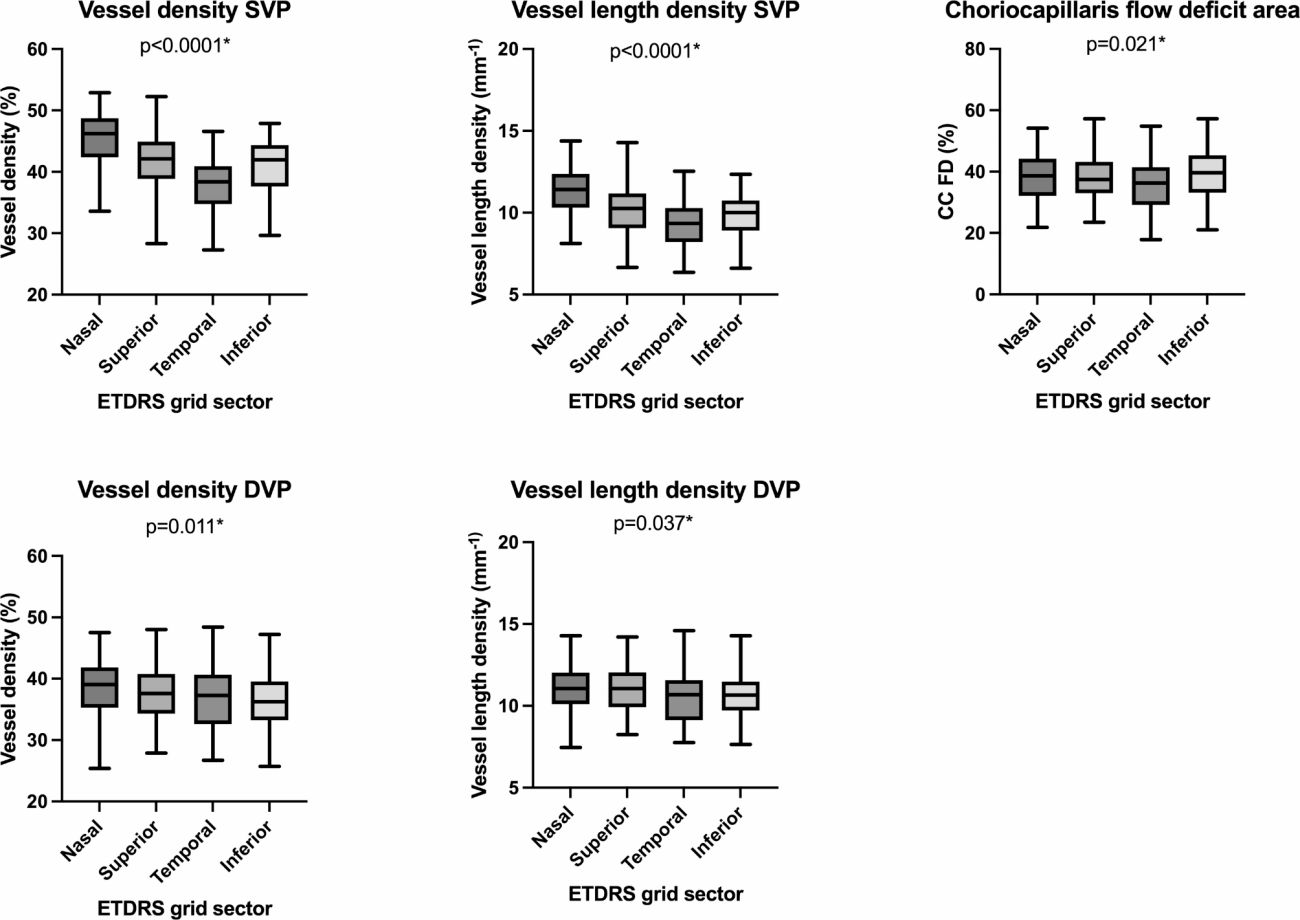
Retinal vascular OCTA metrics in both plexus boxes showing minimum and maximum values, bars median and whiskers range of vessel density and vessel length density in the superficial (SVP) and deep vascular plexus (DVP) in the different sectors of the ETDRS grid: nasal, superior, temporal and inferior. Values of the choriocapillaris flow deficit area (CC FD) are also shown. *P*-values obtained by ANOVA test

To understand the anatomical differences, we compared OCTA metrics in the different sectors of the ETDRS grid centered on fovea. We found significant differences between the different sectors (all *p*-values < 0.04). The temporal sector showed the lowest values for CC FD%, VD and VLD in the SVP. In the DVP, the inferior sector showed lower values (Fig. 2).

**Fig. 3:**
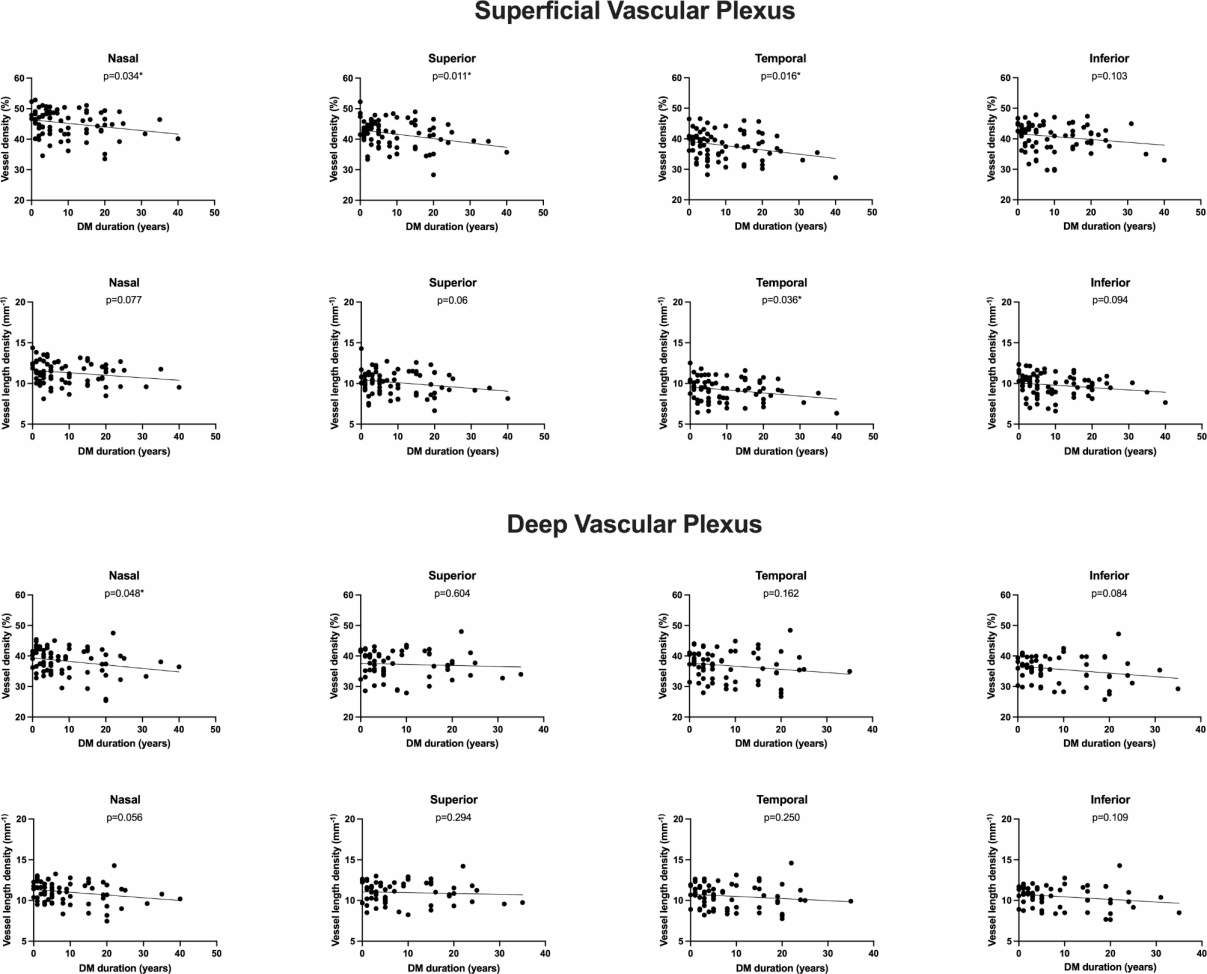
Univariate linear regression between retinal OCTA metrics and disease duration plots show the association between Vessel Density and Vessel length density with the duration of the disease (DM duration), in the different sectors of the ETDRS grid and in the superficial and deep vascular plexuses. *p-value < 0.05

**Fig. 4:**
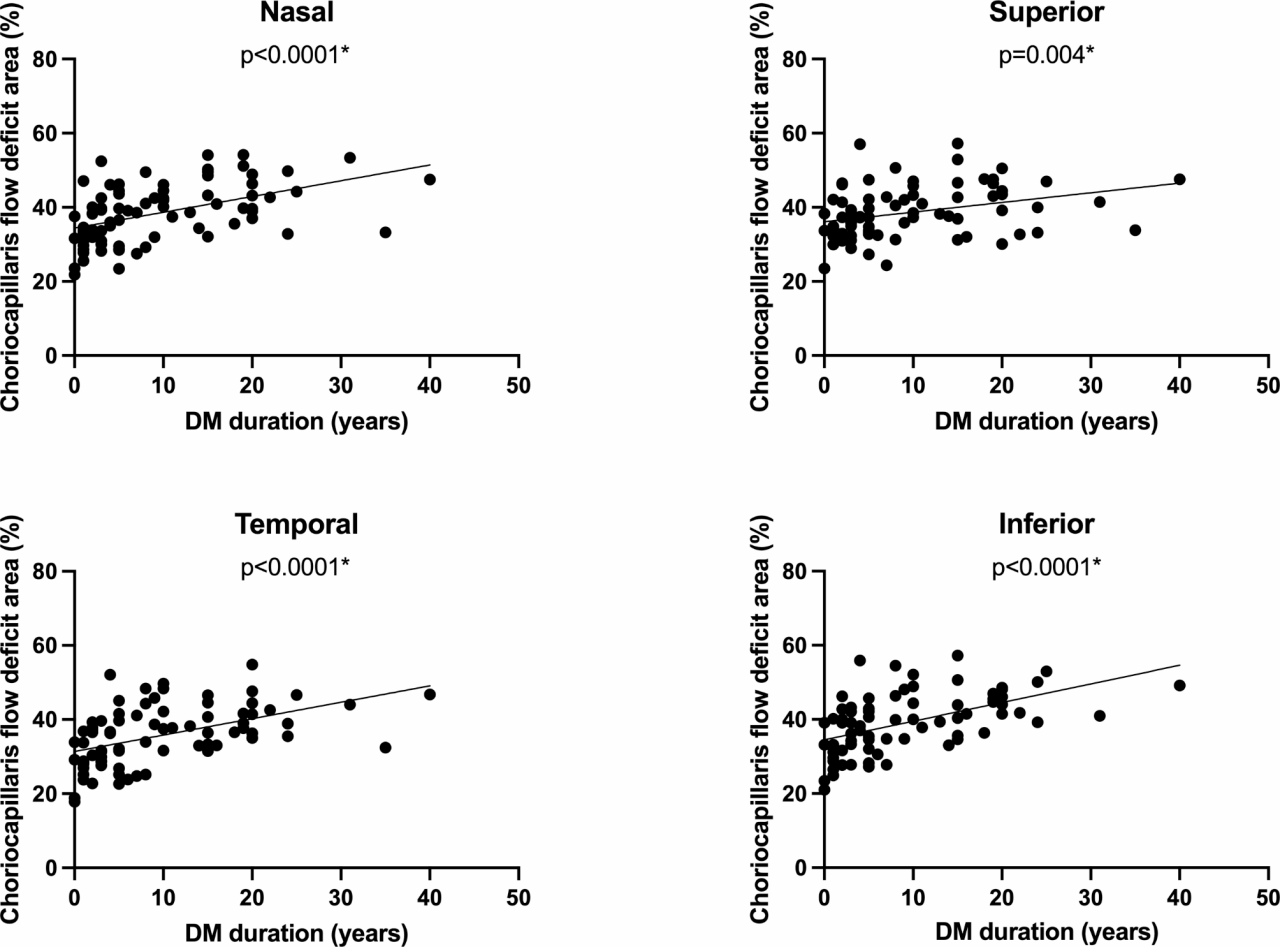
Univariate linear regression between choriocapillaris flow deficit and disease duration plots show the association between choriocapillaris flow deficit area with the duration of the disease (DM duration), in the different sectors of the retina studied. *p-value < 0.05

Patients with longer disease duration had lower VD, VLD and FAZc values and higher CC FD %. Figures 3 and 4 show the linear association between the vascular parameters and the disease duration, with a decrease tendency of VD and VLD in both SVP and DVP and an increase tendency of CC FD% possibly indicating increased ischemia with disease progression.

To study whether the duration of the disease was independently associated with the vascular parameters, we conducted a multivariate analysis adjusting for confounding factors such as age, sex, HbA1c and the presence of arterial hypertension. In this analysis, only CC FD % showed significant association with disease duration in all sectors (all *p* < 0.016). Per each decade, the amount of choriocapillaris avascular area increased by 3.7, 2.3, 3.8 and 4.6% in nasal, superior, temporal and inferior sectors, respectively (Table 2). The VD in the SVP were significant only in the superior and temporal sectors, but showed a lower association. Per each decade, the VD decreased by 1.2 and 1.3% in the superior and temporal sectors, respectively. The rest of the parameters showed no significant association.

**Table 2:** Linear models evaluating the association between diabetes duration and optical coherence tomography angiography parameters

	univariate regression (unadjusted)	multivariate regression (adjusted)
	ß coefficient (95% CI)	
CC FD (%) - Nasal - Superior - Temporal - Inferior	0.43 (0.25 to 0.60)* 0.26 (0.09 to 0.43)* 0.44 (0.26 to 0.62)* 0.50 (0.32 to 0.69)*	0.37 (0.19 to 0.56)* 0.23 (0.04 to 0.42)* 0.38 (0.20 to 0.57)* 0.46 (0.25 to 0.66)*
FAZc	-5.9 (-20.62 to 8.83)	
VD SVP (%) - Nasal - Superior - Temporal - Inferior	-0.12 (-0.23 to -0.01)* -0.14 (-0.25 to -0.03)* -0.14 (-0.26 to -0.03)* -0.09 (-0.21 to 0.02)	-0.10 (-0.22 to 0.02) -0.12 (-0.24 to -0.01)* -0.13 (-0.25 to -0.002)*
VD DVP (%) - Nasal - Superior - Temporal - Inferior	-0.11 (-0.23 to -0.001)* -0.03 (-0.16 to 0.10) -0.10 (-0.25 to 0.04) -0.11 (-0.24 to 0.02)	-0.09 (-0.22 to 0.03)
VLD SVP (mm^− 1^) - Nasal - Superior - Temporal - Inferior	-0.03 (-0.06 to 0.003) -0.03 (-0.07 to 0.001) -0.04 (-0.07 to -0.002)* -0.03 (-0.06 to 0.01)	-0.03 (-0.07 to 0.004)
VLD DVP (mm^− 1^) - Nasal - Superior - Temporal Inferior	0.03 (-0.07 to 0.001) -0.01 (-0.05 to 0.03) -0.03 (-0.07 to 0.02) -0.03 (-0.07 to 0.01)	

Model 2 (multivariate regression) after adjusting by the following confounders: age, sex, HbA1c and presence of arterial hypertension. Choriocapillaris flow deficit percentage (CC FD%), foveal avascular zone circularity (FAZc), vessel density (VD), vessel length density (VLD). SVP: superficial vascular plexus, DVP: deep vascular plexus, *: statistically significant

## Discussion

In this study we show that especially choriocapillaris vascular parameters correlate with T2DM duration and may be explored as candidate biomarkers of retinal health and progression of DR.

Distinct hemodynamic and anatomical characteristics may explain the association of disease duration with choriocapillaris changes. Previous studies indicated that the choroidal flow is regulated by autonomic innervation, yet lacks an intrinsic autoregulatory response and exhibits a relatively poor response to changes in oxygen pressure, in contrast to the retinal circulation [13–15]. Supporting this, Rosen et al. found a transitory increase in retinal perfusion density in diabetic patients before the onset of retinopathy, suggesting a possible autoregulative compensatory mechanism before capillary dropout once DR is established [16]. 

Our results indicate a significant increase in CC flow deficit area of 2.3–4.6% per decade of disease duration, especially in the inferior sector of the ETDRS grid. One possible explanation for the different topographic distribution is the anatomic properties of the choroidal circulation, where some areas are irrigated by end-arteries. These watershed zones are usually located in the inferior sector of the macula, which makes this area more at risk of ischemic damage [17, 18]. In contrast, only two sectors of the retinal parameters (VD in the SVP only) demonstrated significance after adjusting for confounding factors, thereby supporting the notion that choriocapillaris flow behaves in a distinct manner relative to the retinal parameters. Choroidal vascular parameters may serve as early biomarkers of diabetic microvascular damage prior to the onset of retinopathy, offering greater consistency than retinal vascular metrics before clinically visible changes occur.

The predictive value of baseline CC FD% for DR progression has been previously described in recent longitudinal studies [8, 19]. Our results are in line with their findings and support choriocapillaris perfusion as an early sign of ocular vascular impairment in type 2 diabetic patients instead of retinal vascular metrics. We add that CC FD% is independently associated with disease duration by a percentage change of 2.3–4.6% per decade. While CC FD% tends to increase with age [19–21], this is higher than the normal rate of increase per year in healthy subjects, which was reported to be 0.16% in a recent study by Cheng et al. [23] Although the authors used a swept-source OCTA device and therefore baseline measurements may differ from ours, it suggests that the loss of CC FD % expected from normal age changes is less than in patients with T2DM. We included age in the multivariate analysis to account for this.

Ocular anatomy may also explain the topographic differences we found in the mean values of the different retinal sectors examined. The temporal retina is thinner and has a lower metabolic demand and therefore requires less vascular supply, which is supported by previous studies in healthy and diabetic eyes [4, 24, 25]. 

Limitations of this study include the small sample size and a cross-sectional design. Moreover, time to diabetic retinopathy was not available thus limiting our conclusions on the predictive value of OCTA metrics. A common limitation in the OCTA field is the lack of standardization: manufacturers differ in scanning protocols, and image post-processing including binarization techniques which makes comparisons between studies challenging [26]. Moreover, the study population consisted exclusively of patients with type 2 DM. Further investigation is required to ascertain the applicability of our conclusions to a population of type 1 DM. In addition, we only studied an area of 20 × 20º. With the advent of new OCTA protocols that allow ultra-wide field imaging, further investigations are required to examine whether earlier vascular changes occur in the periphery of the retina.

The prospective design and the homogeneous cohort of patients with very good visual acuity, good glycemic control, low refractive errors, and the systematic analysis of 4 different macular areas represent the strengths of our study. Moreover, we included in the analysis the VLD because it is less affected by scanning protocols and postprocessing algorithms than VD, obtaining similar conclusions [27]. 

## Conclusions

Our findings indicate that the percentage of choroidal flow deficit significantly increases with the duration of T2DM in patients without clinically visible retinopathy. This suggests that choroidal parameters are indicative of disease course and may be of value in DR screening and risk stratification.

## Data Availability

The datasets used and/or analysed during the current study are available from the corresponding author on reasonable request.

## Abbreviations

DM: Diabetes mellitus
OCTA: Optical coherence tomography angiography
DR: Diabetic retinopathy
CC FD: Choriocapillaris flow deficit
VD: Vessel density
VLD: Vessel length density
SVP: Superficial vascular plexus
DVP: Deep vascular plexus
FAZc: Foveal avascular zone circularity
T2DM: Type 2 diabetes mellitus
ICP: Intermediate capillary plexus
CC: Choriocapillaris
FAZ: Foveal avascular zone
